# Supplementary material for: A bipartite bacterial virulence factor targets the complement system and neutrophil activation
Source: EMBO J. 2025 Jan 3;44(4):1154–84. doi: 10.1038/s44318-024-00342-8 (PMC11833123; doi:10.1038/s44318-024-00342-8)
Supplement: Supplementary file 1 — Appendix [file 44318_2024_342_MOESM1_ESM.pdf]

Appendix

A bipartite bacterial virulence factor targets the complement system and neutrophil activation

Kurni Kurniyati[1#], Nicholas D. Clark[2#], Hongxia Wang[1], Yijie Deng[1], Ching Wooen Sze[1], Michelle B. Visser[3], Michael G. Malkowski[2*] and Chunhao Li[1*]

Table of Contents

Appendix Table S1 ………………………………………….. Page 2

Appendix Figure S1 …………………………………………. Page 3

Appendix Figure S2 …………………………………………. Page 4

Appendix Figure S3 …………………………………………. Page 4

Appendix Figure S4 …………………………………………. Page 5

Appendix Figure S5 …………………………………………. Page 5

Appendix Figure S6 …………………………………………. Page 6

Appendix Figure S7 …………………………………………. Page 7

Appendix Figure S8 …………………………………………. Page 7

Appendix Figure S9 …………………………………………. Page 8

Appendix Figure S10 ……………………………………….. Page 8

Appendix Figure S11 ………………………………………. Page 9

Appendix Figure S12 ………………………………………. Page 9-10

Appendix Figure S13 ………………………………………. Page 10

References …………………………………………………. Page 10

Appendix Table S1. Crystallographic statistics

Crystallographic Parameter	wild-type C-TDE0362	SeMet C-TDE0362 peak
Space group	$P2_12_12$	$P2_1$
Wavelength (Å)	1.033	0.9794
No. in asymmetric unit	1	4
Unit cell length (Å)		
a	41.60	76.95
b	99.26	91.35
c	121.31	152.13
$\alpha=\gamma$ (°)	90	90
β (°)	90	90.67
Resolution (Å)	39.35 – 1.77	39.26 – 2.19
Highest res. shell (Å)	1.83 – 1.77	2.23 – 2.19
Total observations	418252 (39061)	604557 (29472)
Total unique	49945 (4929)	108136 (5221)
Multiplicity	8.4 (7.9)	5.6 (5.6)
Anom. Multiplicity	N/A	2.8 (2.9)
Completeness (%)	99.9 (99.9)	99.6 (97.4)
Anom. Completeness (%)	N/A	98.4 (94.9)
Mean $I/\sigma(I)$	18.3 (1.8)	13.9 (3.2)
R_{merge} (%)[a, b]	4.88 (82.8)	7.7 (41.2)
$CC^{1/2}$[c]	0.999 (0.894)	0.991 (0.883)
CC^{*}[c]	1 (0.97)	0.99 (0.97)
Anom. CC^{*}[c]	N/A	0.84 (0.42)
Wilson B-factor (Å^2)	32.3	38.31
No. non-hydrogen atoms in refinement	3877	N/A
R_{work}	19.76 (30.26)	N/A
R_{free}[d]	22.58 (32.58)	N/A
Ave. B factor, protein (Å^2)	44.83	N/A
Ave. B factor, solvent (Å^2)	43.89	N/A
Coordinate error (Ǻ)	0.20	N/A
RMSD bond length (Å)	0.007	N/A
RMSD bond angle (°)	0.93	N/A
Ramachandran Plot		
Favored (%)	97.87	N/A

Allowed (%)	2.13	N/A
Outliers (%)	0.00	N/A
Clash score	3.56	N/A

[a] Values in parentheses represent the values in the highest resolution shell.

[b] R_{merge} as defined in (Afonine *et al*, 2012).

[c] $CC^{1/2}$ and CC* as defined in (Karplus & Diederichs, 2012).

[d] 2477 of the reflections (5.1%) were utilized in the test set.

[e] Clash score as calculated in *MOLPROBITY* (Davis *et al*, 2007).

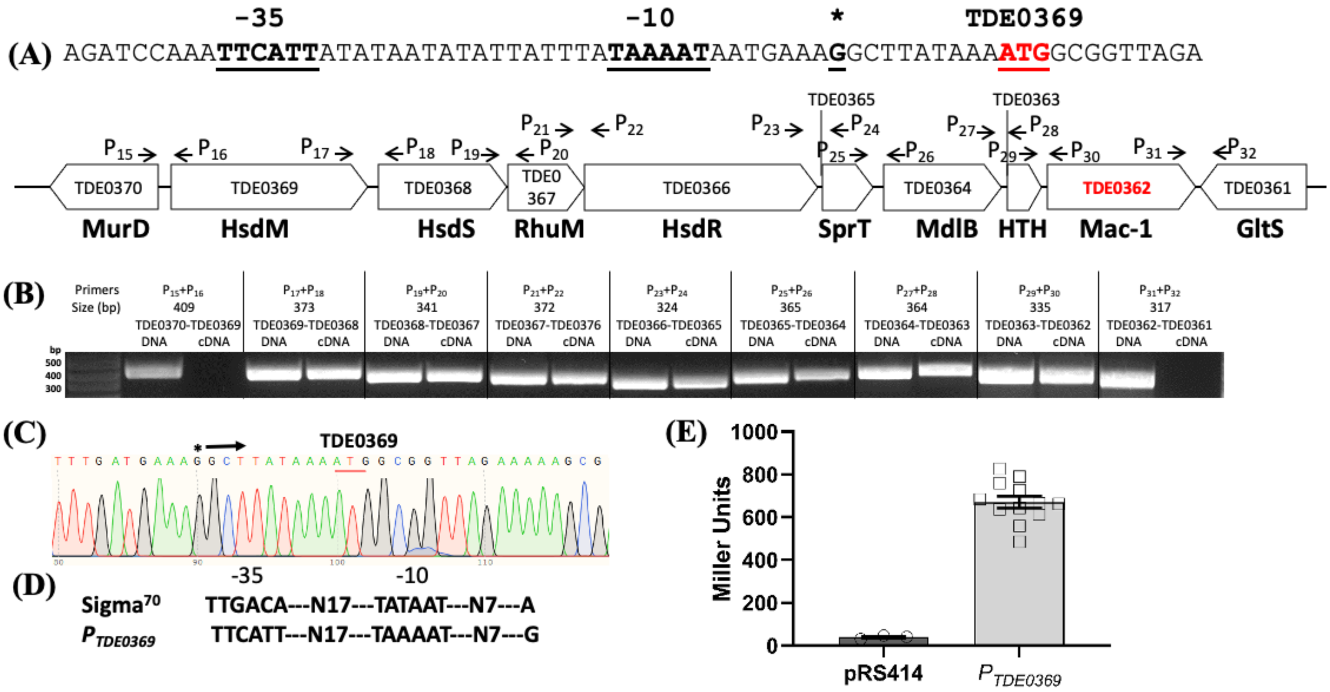

Appendix Figure S1. TDE0362 resides in a gene cluster that is regulated by a sigma[70] -like promoter

(A) Diagram showing the genes adjacent to *TDE0362*. Arrows represent the relative positions and orientations of RT-PCR primers that span the intergenic regions between individual genes as labeled. **(B)** RT-PCR analysis. For each pair of primers, chromosomal DNA (cDNA) was used as a positive control. The numbers below the primers are predicted sizes of RT-PCR and PCR products. **(C)** 5'-RACE analysis. The arrow shows the sequencing direction; asterisk: the transcriptional start site (TSS). The red underlined sequence (ATG) is the start codon of *TDE0369*. **(D)** Sequence comparison between the canonical *E. coli* sigma[70] promoter sequence and the one identified upstream of *TDE0369*, which is designated as $P_{TDE0369}$. **(E)** Transcriptional analysis of $P_{TDE0369}$ using *lacZ* as a reporter in *E. coli*. For this assay, $P_{TDE0369}$ was fused to the promoterless *lacZ* gene in the pRS414 plasmid. The promoterless pRS414 was used as a negative control. β-galactosidase activity was measured and expressed as the average Miller units of triplicate samples from two independent experiments, as previously described (Kurniyati *et al*, 2019). All the primers used here are listed in **Table EV1**.

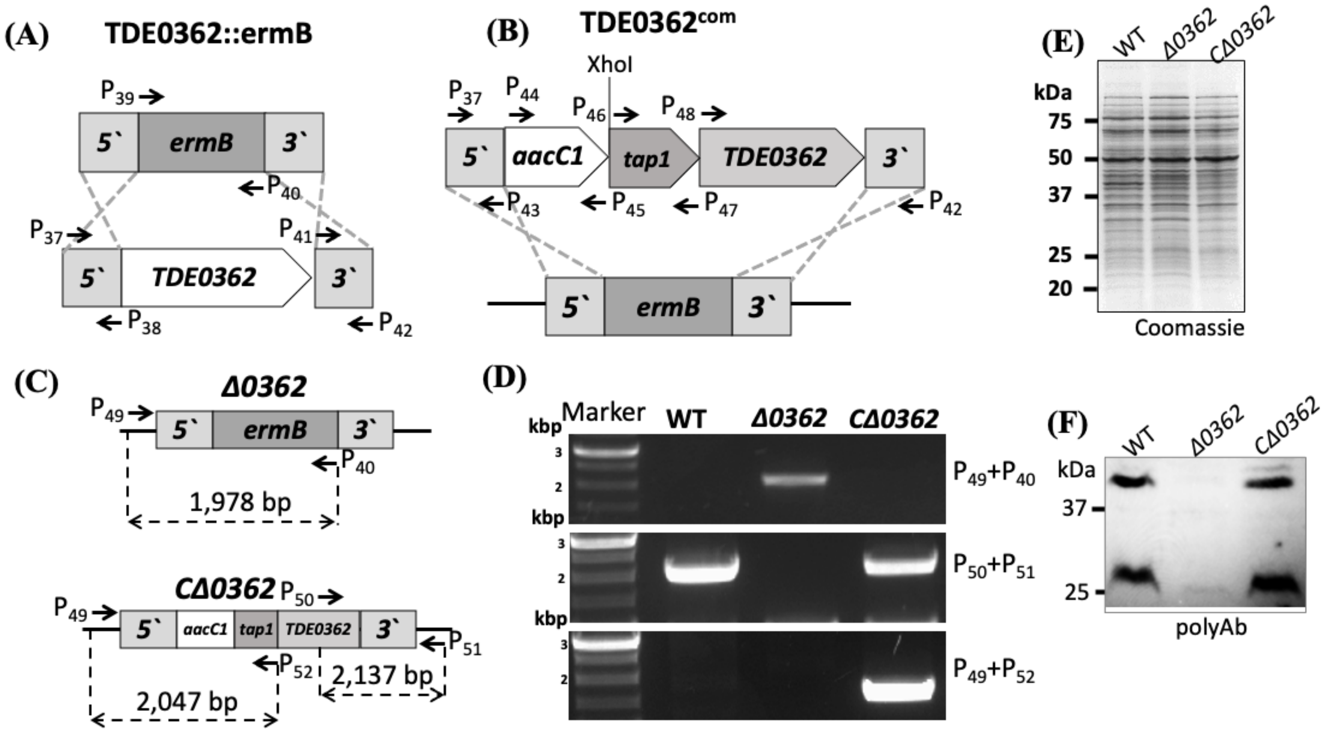

Appendix Figure S2. Construction and characterization of a *TDE0362* deletion mutant (*Δ0362*) and its isogenic *cis*-complemented strain (*CΔ0362*)

(A) TDE0362::ermB was constructed to *in-frame* replace *TDE0362* with the *ermB* cassette.

(B) TDE0362[com] was constructed to replace the *ermB* cassette in the *Δ0362* mutant with *aacC1-tap1-TDE0362*, which was constructed by two-step PCR, as previously described (Bian & Li, 2011).

(C & D) Schematic illustration and PCR analysis of *Δ0362* and *CΔ0362*. Arrows represent the relative positions and orientations of these primers, which are listed in Table EV1. The number of base pairs (bp) are the predicted sizes of PCR products using the corresponding primers as illustrated. *ermB*, an erythromycin B-resistance cassette; *aacC1*, a gentamycin-resistance cassette.

(E & F) Characterization of *T. denticola* WT, *Δ0362*, and C*Δ0362* strains by SDS-PAGE (top panel) and immunoblotting probed against polyAb (lower panel).

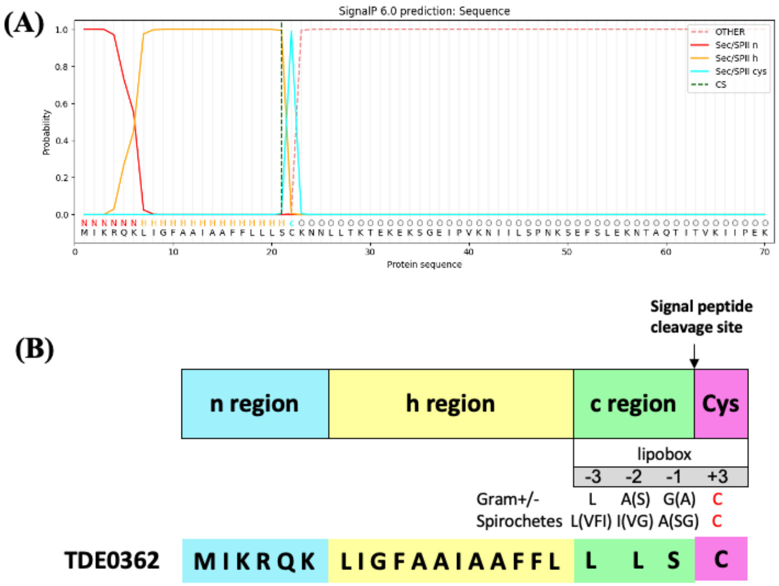

Appendix Figure S3. The N-terminus of TDE0362 contains a lipoprotein signal peptide
(A) Signal peptide prediction analysis using the program SignalIP 6.0 reveals that the N-terminus (1-22 aa) of TDE0362 contains a lipoprotein signal peptide (Sec/SPII) (Probability 0.995171).
(B) A diagram (the top) illustrating a tripartite structure of a lipoprotein signal peptide, including a positively charged N-terminus (n-region), a hydrophobic core in the middle (h region), and a polar region at the carboxyl terminus (c region). The consensus sequences of lipobox in Gram+/- bacteria and spirochetes are also depicted. The bottom illustration depicts the N-terminal sequence of TDE0362, which closely resembles a tripartite structure of a lipoprotein signal peptide.

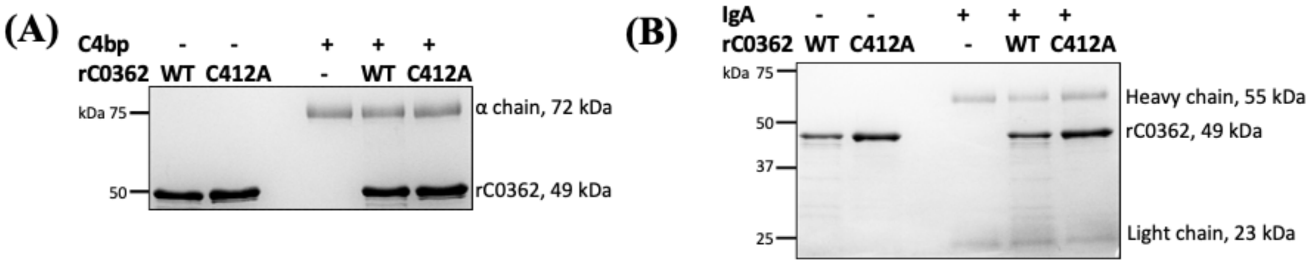

Appendix Figure S4. Measuring the proteolytic activity of recombinant C0362 protein (rC0362) against (A) human C4 binding protein (C4bp) and (B) human IgA.
For this study, C4bp and IgA were incubated with either rC0362 or its inactive form C412A point mutant in a reaction buffer (0.2 M Tris-HCl, pH 7.4, containing 0.1 M NaCl, 5 mM $CaCl_2$ and 2 mM DTT) overnight at 37°C anaerobically. After the incubation, Laemmli sample buffer was added to the samples, boiled for 5 min, and then subjected to SDS-PAGE followed by Coomassie blue staining.

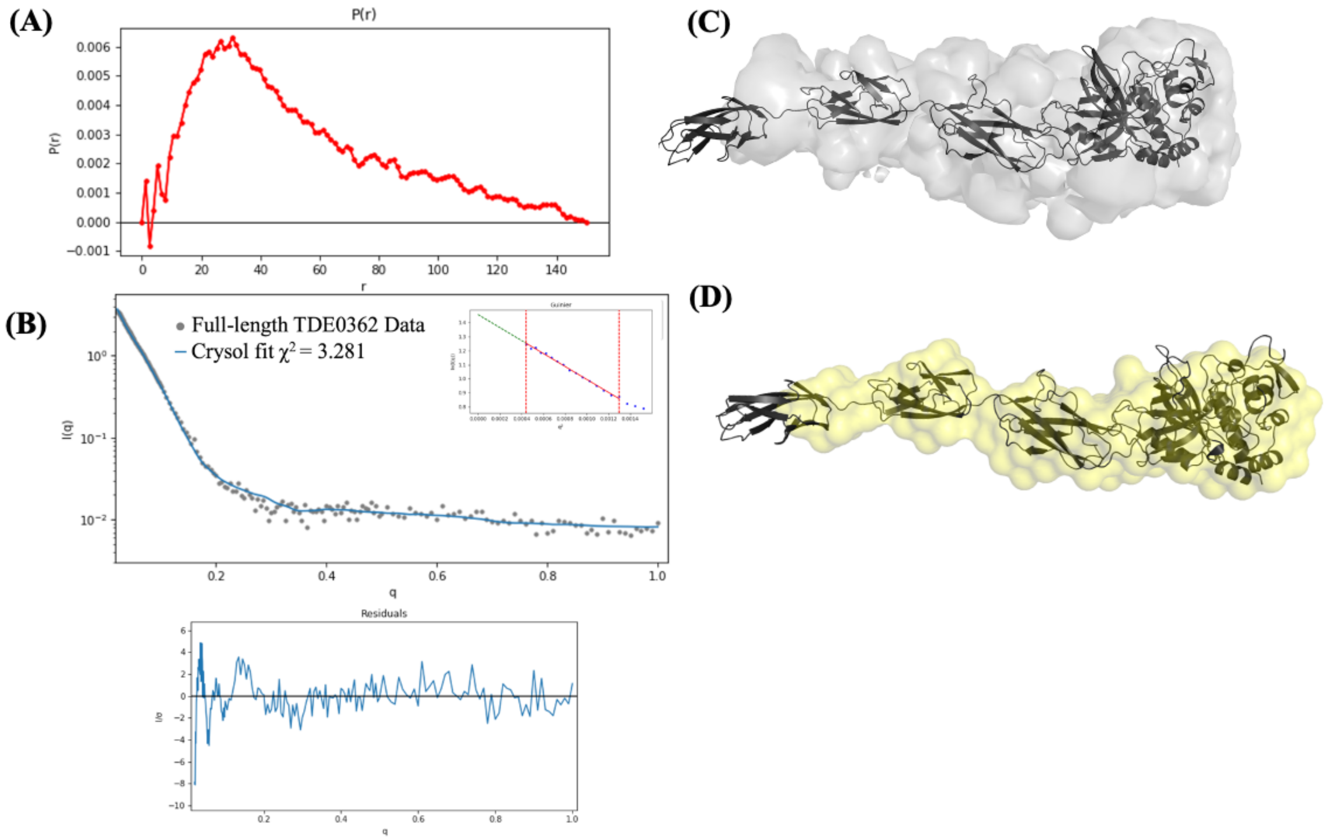

Appendix Figure S5. SEC-SAXS modeling of the full-length TDE0362

(A) Pair-distribution (P(r)) function corresponding to full-length TDE0362 SAXS data suggesting an extended, rod-like shape.

(B) *CRYSOL* fitting of full-length TDE0362 AlphaFold2 model to the experimental SAXS data. Experimental data points are shown as grey dots, while the *CRYSOL* calculated SAXS profile of the full-length TDE0362 model is shown as a blue line. The chi-squared value of 3.281 for the fit indicates the AlphaFold2 model is representative of the experimental data. Shown below is the residuals plot. The inset shows the Guinier fit of the experimental SAXS data.

(C) Full-length TDE0362 secondary structure is shown as a black cartoon and fits well to the SAXS-derived electron density shown as a grey volume.

(D) The *DAMIF/DAMIN* bead model molecular envelope shown as a yellow surface (4.5Å bead radius).

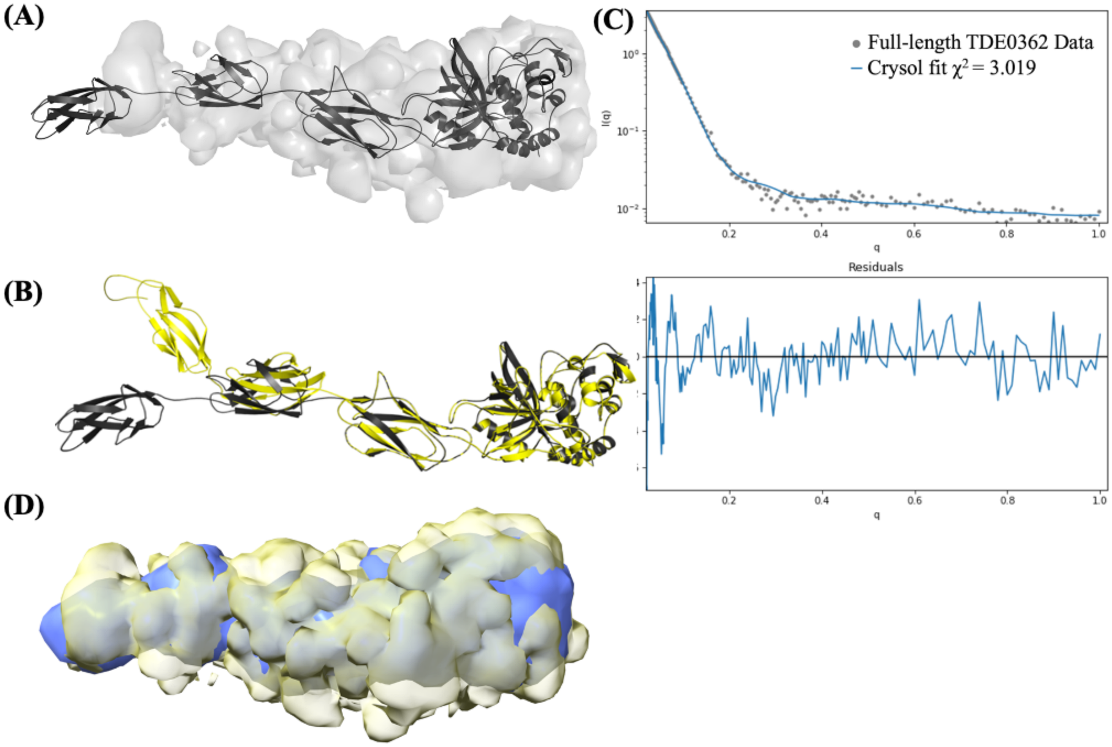

Appendix Figure S6. Fitting of the full-length TDE0362 model to the SEC-SAXS data

(A) The DENSS electron density map is shown as a grey volume, contoured at 3$\boldsymbol{\sigma}$. Even at this tighter cutoff, the modified model (black cartoon) fits well to the DENSS electron density map envelope.

(B) Shown is the alignment of the full-length TDE0362 AlphaFold2 model (yellow cartoon) superimposed onto the modified model (black cartoon), which was modified to fit the *DENSS* electron density reconstruction and *DAMMIF/DAMMIN* bead model.

(C) Assessment of the modified AlphaFold2 model fit to the raw SEC-SAXS data by *CRYSOL* shows a high correlation (chi-squared of 3.019). Raw SAXS data is shown as grey dots and the *CRYSOL* calculated SAXS profile of the model is shown as a blue line. Residuals for the *CRYSOL* fit are shown below.

(D) Overlay of the *DENSS* electron density map (yellow, transparent volume) and the molecular envelope from the *DAMMIF/DAMMIN* bead modeling. Despite some minor differences, the two envelopes are in close agreement.

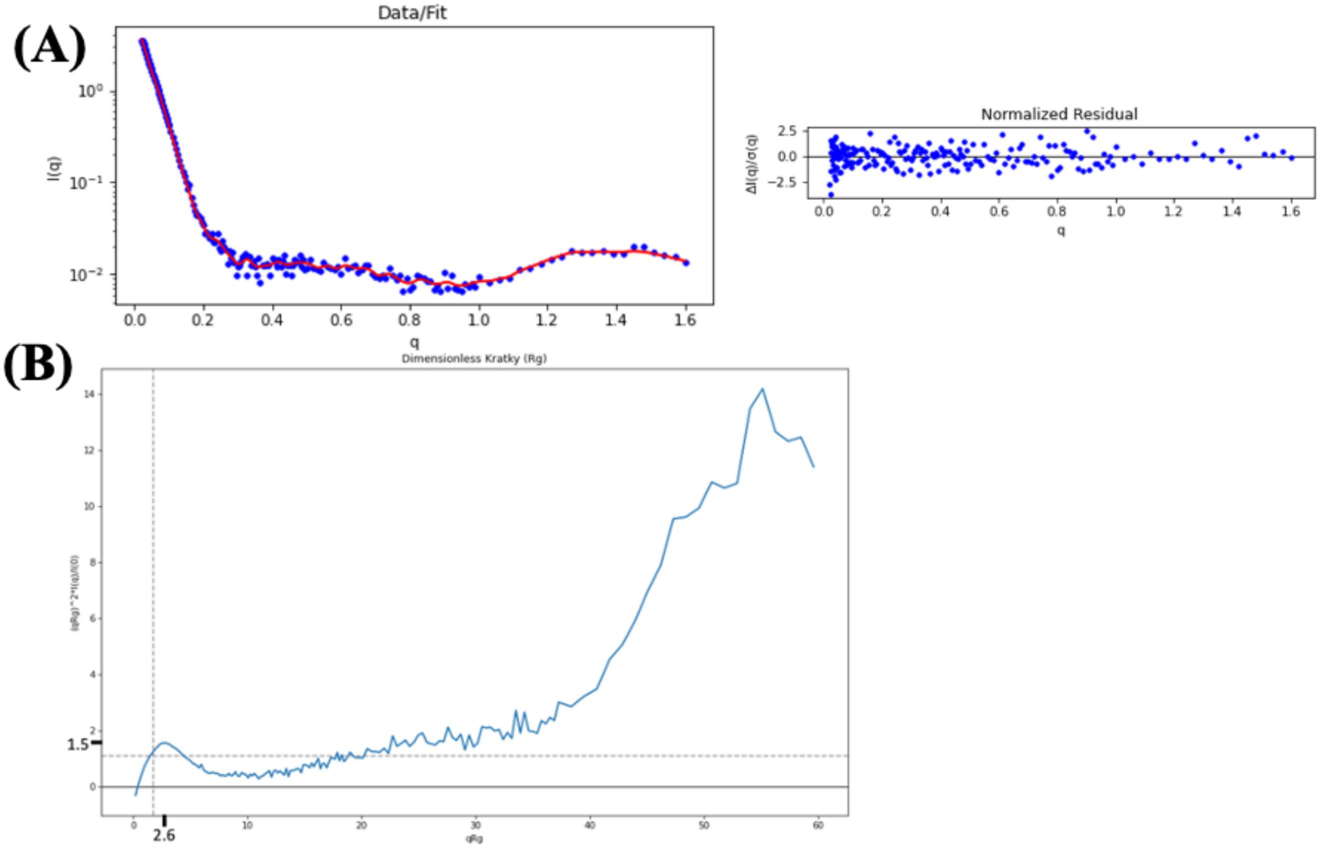

Appendix Figure S7. SEC-SAXS analysis of the full-length TDE0362
(A) Indirect Fourier Transform (IFT; red line) fit of the full-length TDE0362 SEC-SAXS data (blue dots) over a q-range of 0.021-1.6 1/Å. Residuals of the fit are shown on the right.
(B) Shown here is the dimensionless Kratky plot for the IFT fit data in (A) which we used to qualitatively assess the flexibility and folding of full-length TDE0362. Our peak at 2.66, 1.55 suggests a "partial folding" or highly flexible TDE0362, with the latter more likely. This is supported by the same analysis using the rigid AlphaFold model (i.e., completely folded) to directly generate a dimensionless Kratky plot yielding a similar shape and peak. Guidelines for a globular protein (qRg=1.73, height=1.11) are shown as grey dashed lines.

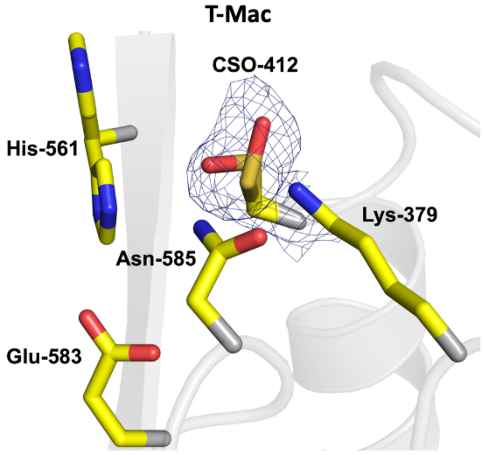

Appendix Figure S8. Electron density corresponding to CSO-412. Depicted is the active-site architecture of the Mac-1 domain of C-TDE0362. Additional electron density was observed off the S_γ atom of Cys-412, which was best fit when modeled as a cysteine sulfonic acid (CSO), corresponding to a single oxidation of the sulfur atom. $2mF_o$-DF_c, contoured at $1\boldsymbol{\sigma}$, electron density is shown as blue mesh and fits well to the modeled CSO residues.

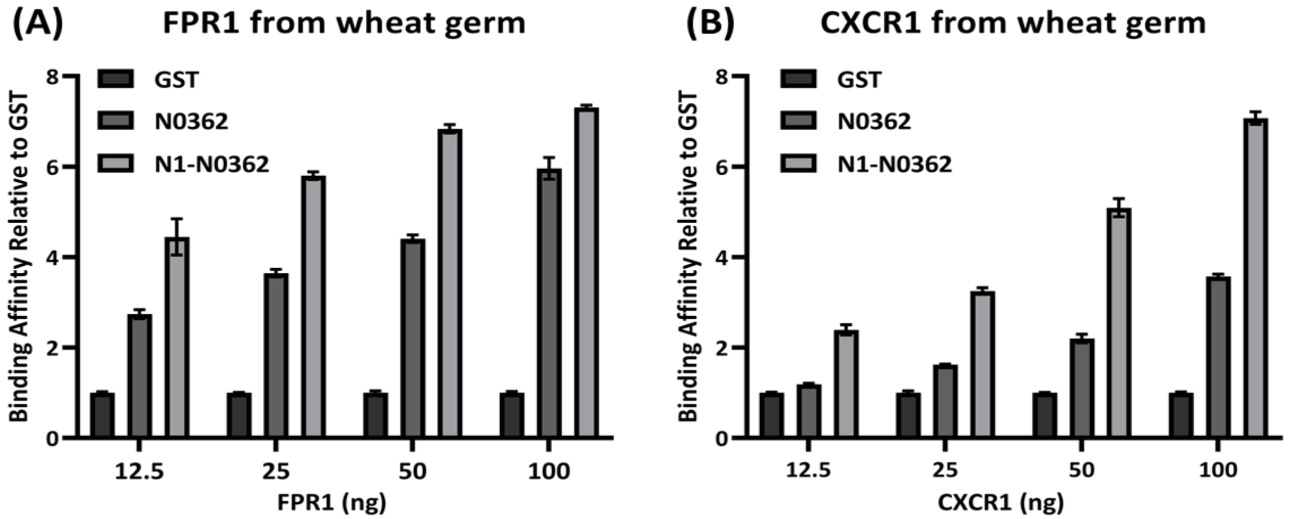

Appendix Figure S9. Measuring the binding between N0362 and FPR1 and CXCR1 receptors using enzyme-linked immunosorbent assays (ELISA)

This study was performed by immobilizing GST, N0362 (aa 23-204) and N1-N0362 (aa 23-114) on high binding microtiter wells, followed by incubation with increasing amounts of either FPR1 or CXCR1, ranging from 12.5 to 100 µg of total proteins. The interactions were detected using a polyclonal antibody for GST, followed by incubation with a horseradish peroxidase-conjugated sheep anti-goat IgG (H+L). The binding was measured by the absorbance at 450 nm of the enzymatic conversion of 3,3',5,5'-tetramethylbenzidine. The data are presented as the mean of binding affinity relative to GST ± standard error of the mean (SEM).

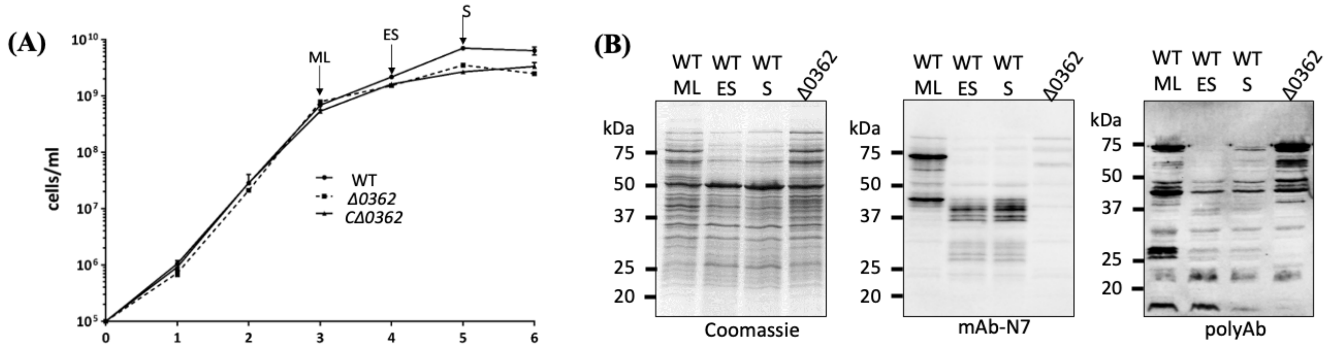

Appendix Figure S10. Detection of TDE0362 at different growth phases of *T. denticola* by immunoblotting analysis

(A) Measuring the growth curve of WT, *Δ0362*, and *CΔ0362* strains. A total of 1×10^5 cells/ml of late-log phase *T. denticola* cultures was inoculated into the TYGVS medium. Cells were enumerated every 24 hours using a Petroff Hausser counting chamber. Samples were harvested at three different growth phases as indicated and subjected to **(B)** immunoblotting analysis using polyAb and mAb-N7, a monoclonal antibody against TDE0362. ML: middle log phase; ES: early stationary phase; S: stationary phase.

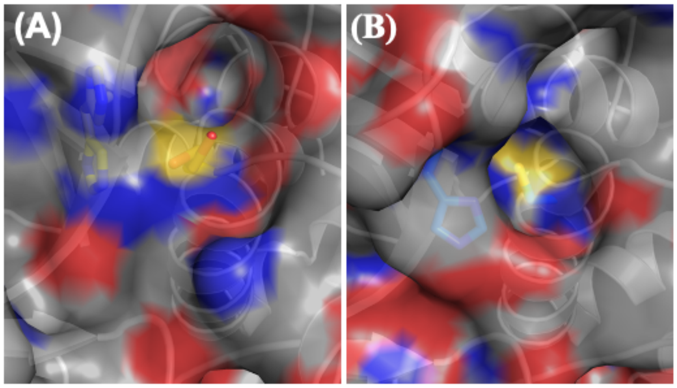

Appendix Figure S11. Active site surface comparison of C0362 and gasMac-1

(**A**) Surface representation of C0362 active site. Comparison of the active site surfaces of C0362 and gasMac-1 shows that C0362 has a more open cleft than in gasMac-1. Surface shows carbon atoms colored grey, while oxygen and nitrogen atoms are colored in red and blue, respectively. The active site Cys412 and His561 are shown as yellow sticks, with oxygen and nitrogen atoms colored red and blue, respectively.

(**B**) Surface representation of gasMac-1 (PDB entry 2AU1). Catalytic Cys94 and His262 are shown as blue sticks, with oxygen and nitrogen atoms colored red and blue, respectively.

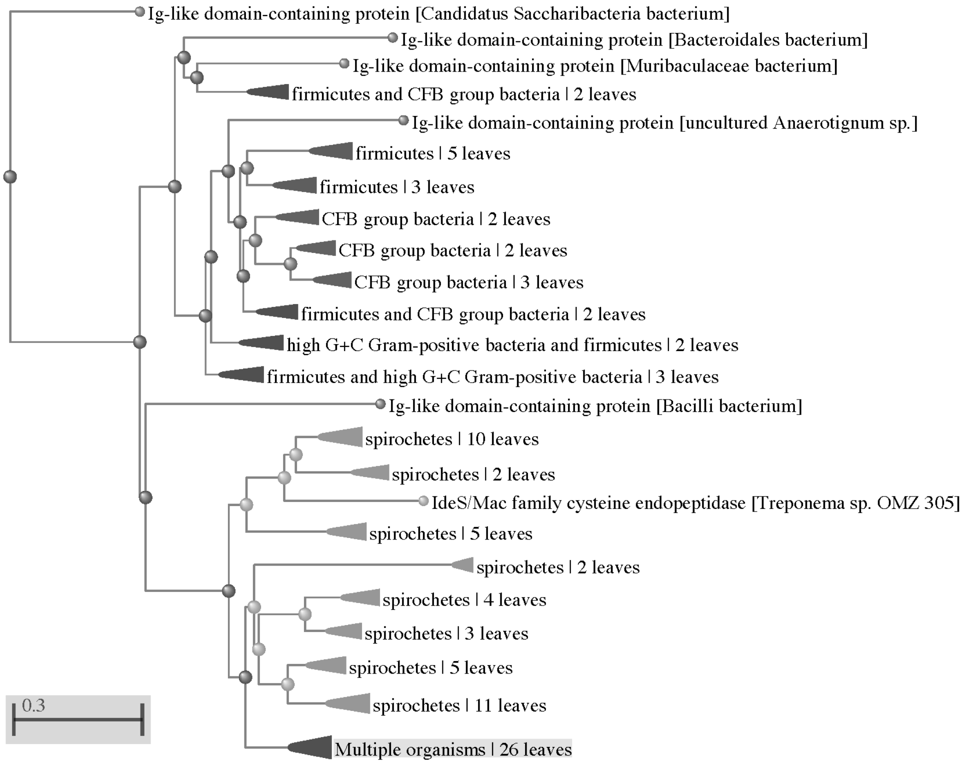

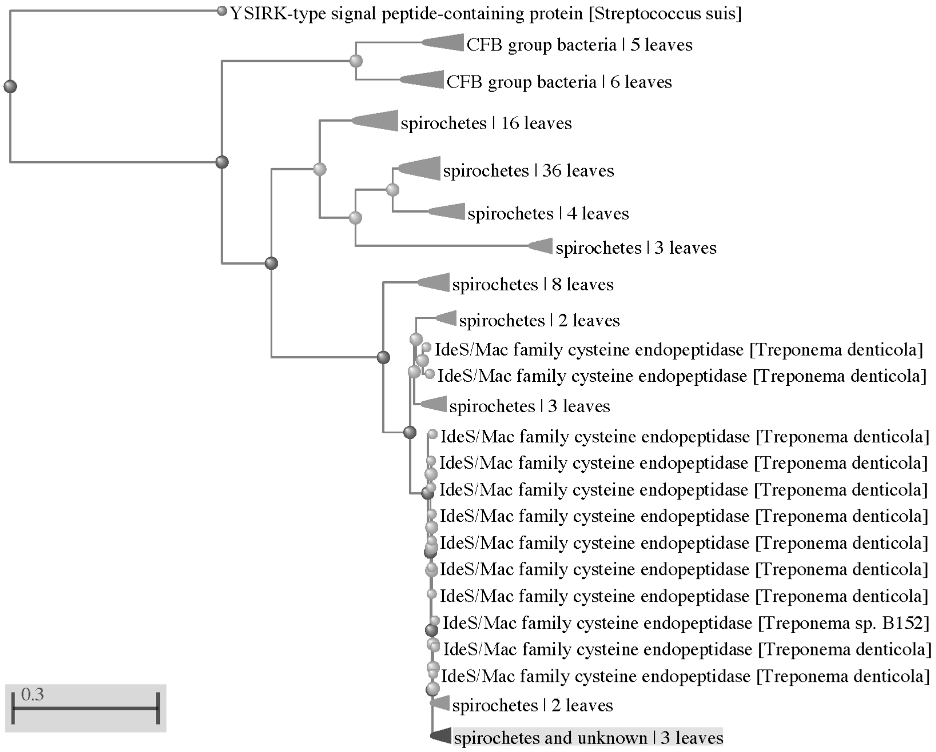

Appendix Figure S12. Phylogenetic analysis of TDE0362 homologs

For this study, we used the Big domains in TDE0362 (top panel) and the C-terminal Mac-1 domain (lower panel) as a query to the search bacterial genomes in NCBI and distant trees were generated using BLAST pairwise alignments.

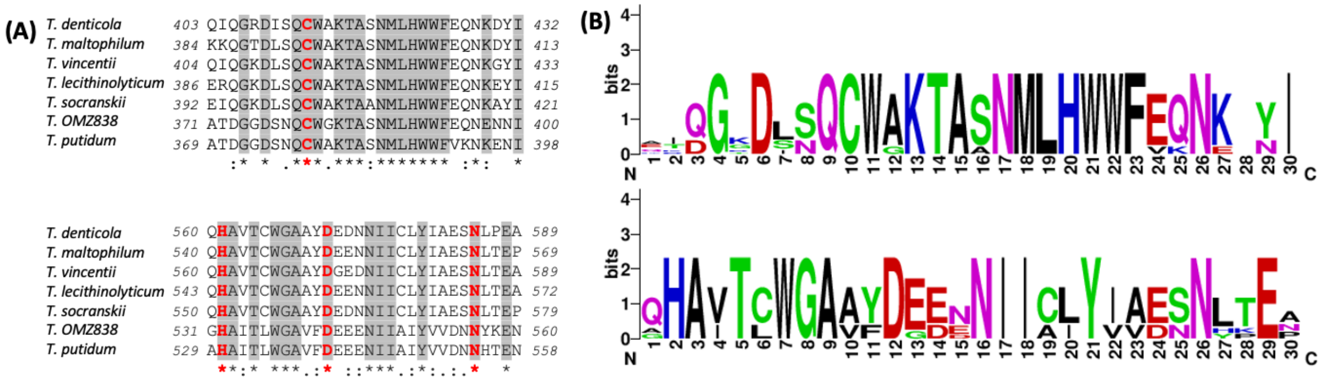

Appendix Figure S13. Multiple sequence alignments of TDE0362 homologs from different oral Treponemes

Multiple sequence alignments were conducted using **(A)** Clustal Omega **(B)** Sequence Logos. The numbers show the positions of amino acids of *T. denticola* TDE0362 (AAS10857), *Treponema maltophilum* (WP_314743588), *Treponema vincentii* (WP_162662933), *Treponema lecithinolyticum* (WP_314743588), *Treponema socranskii* (WP_315327333), *Treponema sp. OMZ 838* (WP_052185531), and *Treponema putidum* (WP_255819007). Only the conserved region in C-terminal cysteine protease domains was listed here.

References

Afonine PV, Grosse-Kunstleve RW, Echols N, Headd JJ, Moriarty NW, Mustyakimov M, Terwilliger TC, Urzhumtsev A, Zwart PH, Adams PD (2012) Towards automated crystallographic structure refinement with phenix.refine. *Acta Crystallogr D Biol Crystallogr* 68: 352-367

Bian J, Li C (2011) Disruption of a type II endonuclease (TDE0911) enables Treponema denticola ATCC 35405 to accept an unmethylated shuttle vector. *Appl Environ Microbiol* 77: 4573-4578

Davis IW, Leaver-Fay A, Chen VB, Block JN, Kapral GJ, Wang X, Murray LW, Arendall WB, 3rd, Snoeyink J, Richardson JS *et al* (2007) MolProbity: all-atom contacts and structure validation for proteins and nucleic acids. *Nucleic Acids Res* 35: W375-383

Karplus PA, Diederichs K (2012) Linking crystallographic model and data quality. *Science* 336: 1030-1033

Kurniyati K, Liu J, Zhang JR, Min Y, Li C (2019) A pleiotropic role of FlaG in regulating the cell morphogenesis and flagellar homeostasis at the cell poles of Treponema denticola. *Cell Microbiol* 21: e12886
