# Supplementary material for: A bipartite bacterial virulence factor targets the complement system and neutrophil activation
Source: EMBO J. 2025 Jan 3;44(4):1154–84. doi: 10.1038/s44318-024-00342-8 (PMC11833123; doi:10.1038/s44318-024-00342-8)
Supplement: Supplementary file 2 — Table EV1 [file 44318_2024_342_MOESM2_ESM.pdf]

**Table EV1. Oligonucleotide primers used in this study**

| Primers | Sequences (5'-3') | Note [a] |
|---|---|---|
| $P_1$ | GGATCCACCGAAAAAGAGAAATCCGG | FL0362 recombinant protein; [F] |
| $P_2$ | CTGCAGTTACTGATGTGCCTCAAGCC | FL0362 recombinant protein; [R] |
| $P_3$ | GGATCCTGTAAAAACAATCTTTTAAC | N0362, yeast expression using p426TEF; [F] |
| $P_4$ | GTCGACTTATTTGGCTACCGAGGGAGTGGAG | N0362, yeast expression using p426TEF; [R] |
| $P_5$ | GGATCCAAAAACAACCTGCTGACCAAAAC | N1-N0362 recombinant protein; [F] |
| $P_6$ | AAGCTTCTACTTCTTCACCCCATTCGAAGC | N1-N0362 recombinant protein; [R] |
| $P_7$ | GGATCCACGATAGACGTTATCGTAAC | N2-N0362 recombinant protein; [F] |
| $P_8$ | AAGCTTTTTGGCTACCGAGGGAGTGG | N2-N0362 recombinant protein; [R] |
| $P_9$ | CACCAAAAACAATCTTTTAACAAAAAC | N0-N0362 recombinant protein; [F] |
| $P_{10}$ | TTATTTGGCTACCGAGGGAGTGGAG | N0-N0362 recombinant protein; [R] |
| $P_{11}$ | CACCATGACGGTTACAATTGAAATTAAAAAG | C0362 recombinant protein; [F] |
| $P_{12}$ | CTGATGTGCCTCAAGCCACTTTTTC | C0362 recombinant protein; [R] |
| $P_{13}$ | GGATCCATGATAAAAAGACAGAAATT | FL0362, yeast expression using p426GPD; [F] |
| $P_{14}$ | GTCGACCTGATGTGCCTCAAGCCAC | FL0362 and C0362, yeast expression using p426GPD; [R] |
| $P_{15}$ | GATTGACGGCCTTAGCTCTTC | Co-RT-PCR, *TDE0370*; [R] |
| $P_{16}$ | CTGAGAGCTGTCCATCCCGCC | Co-RT-PCR, *TDE0369*; [R] |
| $P_{17}$ | CATCATCTGTCCGAACGG | Co-RT-PCR, *TDE0369*; [F] |

| | | |
|---|---|---|
| $P_{18}$ | CCTTGCCCTGCAAGACCTAC | Co-RT-PCR, *TDE0368*; [R] |
| $P_{19}$ | GGGCAACTGTTGATATGGC | Co-RT-PCR, *TDE0368*; [F] |
| $P_{20}$ | GATATGCCGTGAAATAGTAG | Co-RT-PCR, *TDE0367*; [R] |
| $P_{21}$ | GAACAGCTTGATGCAGTATTG | Co-RT-PCR, *TDE0367*; [F] |
| $P_{22}$ | GAAGAAGTCAGCCAAGTGAG | Co-RT-PCR, *TDE0366*; [R] |
| $P_{23}$ | GCTCTTACCATTGATAAG | Co-RT-PCR, *TDE0366*; [F] |
| $P_{24}$ | CCAAGATAGTTTTGTTCG | Co-RT-PCR, *TDE0365*; [R] |
| $P_{25}$ | CCGGAATGTTTGGAATATGTC | Co-RT-PCR, *TDE0365*; [F] |
| $P_{26}$ | CTTAACAGAAAAGGTACAAC | Co-RT-PCR, *TDE0364*; [R] |
| $P_{27}$ | CAGATGCAGAAGTTTTAATC | Co-RT-PCR, *TDE0364*; [F] |
| $P_{28}$ | GTAAAACGCCACTGAGAACC | Co-RT-PCR, *TDE0363*; [R] |
| $P_{29}$ | GAGAAAGCAAATAAATGCCG | Co-RT-PCR, *TDE0363*; [F] |
| $P_{30}$ | CGGGGATTTCACCGGATTTC | Co-RT-PCR, *TDE0362*; [R] |
| $P_{31}$ | CATCTATATTGATGGCCTTAC | Co-RT-PCR, *TDE0362*; [F] |
| $P_{32}$ | GCAACCCCTAACGCCATGGC | Co-RT-PCR, *TDE0361*; [F] |
| $P_{33}$ | GCAAGCATATCATCAAAGCTGC | *TDE0369* 5`RACE outer primer; [R] |
| $P_{34}$ | CACCGTAGGGCATATCTTTG | *TDE0369* 5`RACE inner primer; [R] |
| $P_{35}$ | GAATTCAGCATCATTATAACAAAAAATT | *TDE0369* promoter for pRS414; [F] |

| | | |
|---|---|---|
| $P_{36}$ | GGATCCGCCCACAAACTACCATATAA | *TDE0369* promoter for pRS414; [R] |
| $P_{37}$ | GCGACATTGTTTGGGTACAGG | 5` portion for TDE0362 inactivation; [F] |
| $P_{38}$ | GAATATTTTATATTTTTGTTCATAAAATAATC TCCAATATGTT | 5` portion for TDE0362 inactivation; [R] |
| $P_{39}$ | ATGAACAAAAATATAAAATATTCTC | Erythromycin B (*ermB*) cassette; [F] |
| $P_{40}$ | TTATTTCCTCCCGTTAAATAATAG | Erythromycin B (*ermB*) cassette; [R] |
| $P_{41}$ | TATTTAACGGGAGGAAATAAGCCGACAATA AAGGGCTTGG | 3` portion for TDE0362 inactivation; [F] |
| $P_{42}$ | GCACCGATTGTTGATCCGGA | 3` portion for TDE0362 inactivation; [R] |
| $P_{43}$ | CGTTGCTGCTGCGTAACATAAAATAATCTCC AATATGTT | 5` portion for TDE0362 complementation; [R] |
| $P_{44}$ | AACATATTGGAGATTATTTTATGTTACGCAG CAGCAACG | Gentamicin (*aacC1*) cassette for TDE0362 complementation; [F] |
| $P_{45}$ | CTCGAGGCCGACAATAAAGGGCTTGG | Gentamicin (*aacC1*) cassette for TDE0362 complementation; [R] |
| $P_{46}$ | CTCGAGATTATACTTCTCCTTAAAAC | *tap1* promoter for TDE0362 complementation; [F] |
| $P_{47}$ | AATTTCTGTCTTTTTATCATATGAACCTCC | *tap1* promoter for TDE0362 complementation; [R] |
| $P_{48}$ | AAGTTTTTATGGAGGTTCATATGATAA | *TDE0362* for TDE0362 complementation; [F] |
| $P_{49}$ | GAGACAATCAGATCGATTG | Flanking region of *TDE0362* for PCR analysis; [F] |
| $P_{50}$ | GGATCCGTCGATGGAATAGGAGCACC | *TDE0362* for PCR analysis; [F] |
| $P_{51}$ | CTCGCCGTCATGCTGGGTTC | Flanking region of *TDE0362* for PCR analysis; [R] |
| $P_{52}$ | ATGAACCTCCATAAAAAC | *tap1* promoter for PCR analysis; [R] |
| $P_{53}$ | GGATCCGTCGATGGAATAGGAGCACC | C0362, yeast expression using p426GPD; [F] |

| | | |
|---|---|---|
| $P_{54}$ | GGATCCAGAACACAATGTCCATAAAAAGACAGAAATTAATAG | FL0362, yeast expression using pYES2/NTA; [F] |
| $P_{55}$ | TCTAGACTGATGTGCCTCAAGCCAC | FL0362 and C0362, yeast expression using pYES2/NTA; [R] |
| $P_{56}$ | GGATCCAGAACACAATGTCCGTCGATGGAATAGGAGCACC | C0362, yeast expression using pYES2/NTA; [F] |
| $P_{57}$ | GGGCGAGATATTAGCCAAGCCTGGGCAAAAACCGCTTC | C412A site directed mutagenesis; [F] |
| $P_{58}$ | GAAGCGGTTTTTGCCCAGGCTTGGCTAATATCTCGCCC | C412A site directed mutagenesis; [R] |
| $P_{59}$ | GCCATACCAAGCTGCCGTAACG | H561A site directed mutagenesis; [F] |
| $P_{60}$ | CTGTTACCTTTAGAACCC | H561A site directed mutagenesis; [R] |
| $P_{61}$ | GCAGCCTATGCCGAAGATAATAATATTATCTGTCTC | D571A site directed mutagenesis; [F] |
| $P_{62}$ | GCCCCAACACGTTACGGC | D571A site directed mutagenesis; [R] |
| $P_{63}$ | TATATTGCCGCATCAAACTTACC | E583A site directed mutagenesis; [F] |
| $P_{64}$ | GAGACAGATAATATTATTATCTTCG | E583A site directed mutagenesis; [R] |
| $P_{65}$ | CTATATTGCCGAATCAGCCTTACCGGAAGCTGTC | N585A site directed mutagenesis; [F] |
| $P_{66}$ | GACAGCTTCCGGTAAGGCTGATTCGGCAATATAG | N585A site directed mutagenesis; [R] |
| $P_{67}$ | TAATGTGCGGGCATTAGATAAGTTG | K379A site directed mutagenesis; [F] |
| $P_{68}$ | TAAAATTTTGTATCGGCAGTTTC | K379A site directed mutagenesis; [R] |
| $P_{69}$ | AGCTGTCCTCGCTCCATTCGGC | Y592A site directed mutagenesis; [F] |
| $P_{70}$ | TCCGGTAAGTTTGATTCGGC | Y592A site directed mutagenesis; [R] |
| $P_{71}$ | CCTCTATCCAGCCGGCGTTAGATAC | K594A site directed mutagenesis; [F] |

| | | |
|---|---|---|
| $P_{72}$ | ACAGCTTCCGGTAAGTTTGATTCG | K594A site directed mutagenesis; [R] |
| $P_{73}$ | GCCATGGCCATCAAGC GGCAGAAGCTGATC | N0362, mammalian expression; [F] |
| $P_{74}$ | CTTGTCGTCATCGTCCTTGTAGTCCTTGGCCACGCTAGGGGTGG | N0362, mammalian expression; [R] |
| $P_{75}$ | GCCATGGCCGTCGACGGCATCGGCGCCCC | C0362, mammalian expression; [F] |
| $P_{76}$ | CTTGTCGTCATCGTCCTTGTAGTCCTGGTGGGCTTCCAGCCATTTC | C0362, mammalian expression; [R] |
| $P_{77}$ | CAGAGATATCAGCCAAGCTTGGGCCAAGACCG | Codon optimized C412A for mammalian expression; [F] |
| $P_{78}$ | CCTTGGATCTGCTCATTTCTG | Codon optimized C412A for mammalian expression; [R] |
| $P_{79}$ | GGATCCATGTCAAATATAACAGATCC | CXCR1 recombinant protein; [F] |
| $P_{80}$ | GTCGACCAGATTTGAGCTGACATTAAC | CXCR1 recombinant protein; [R] |

[a] Underlined sequences are engineered restriction cut sites for DNA cloning; [F] forward; [R] reverse.
