# Supplementary figures and images for: A bipartite bacterial virulence factor targets the complement system and neutrophil activation
Source: EMBO J. 2025 Jan 3;44(4):1154–84. doi: 10.1038/s44318-024-00342-8 (PMC11833123; doi:10.1038/s44318-024-00342-8)

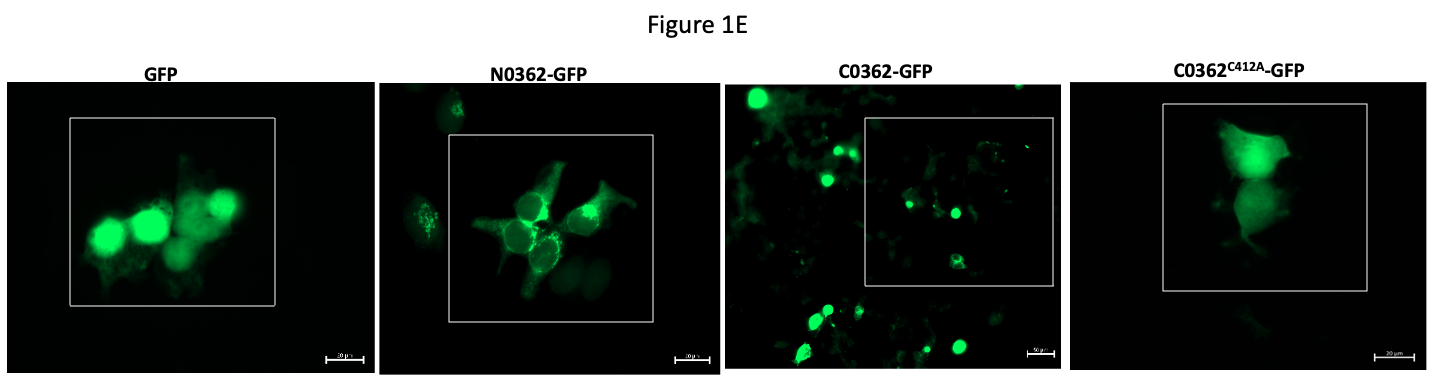

Supplement: Supplementary file 4 — Source data Fig. 1 [file 44318_2024_342_MOESM4_ESM.zip › Figure 1/Figure 1E.tiff]

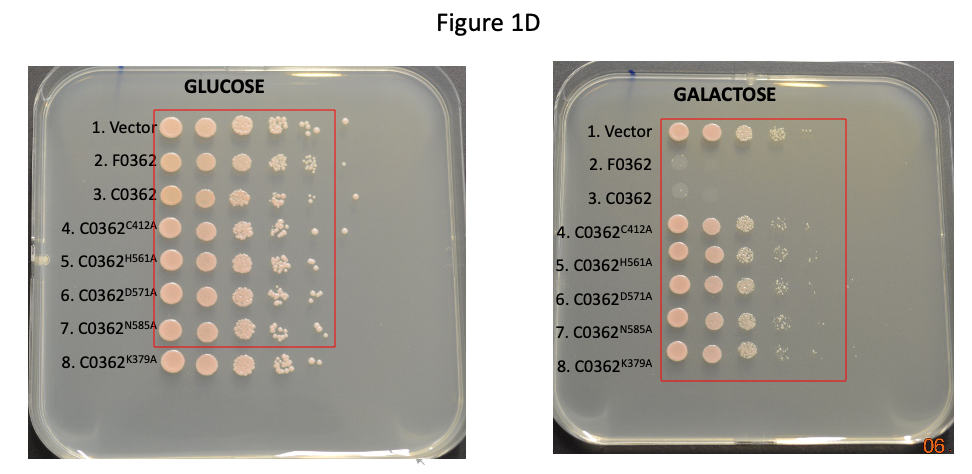

Supplement: Supplementary file 4 — Source data Fig. 1 [file 44318_2024_342_MOESM4_ESM.zip › Figure 1/Figure 1D.tiff]

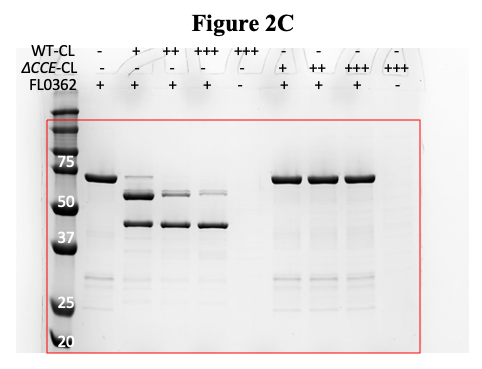

Supplement: Supplementary file 5 — Source data Fig. 2 [file 44318_2024_342_MOESM5_ESM.zip › Figure 2/Figure 2C.tiff]

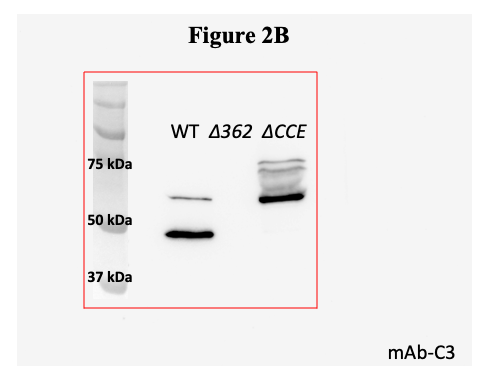

Supplement: Supplementary file 5 — Source data Fig. 2 [file 44318_2024_342_MOESM5_ESM.zip › Figure 2/Figure 2B.tiff]

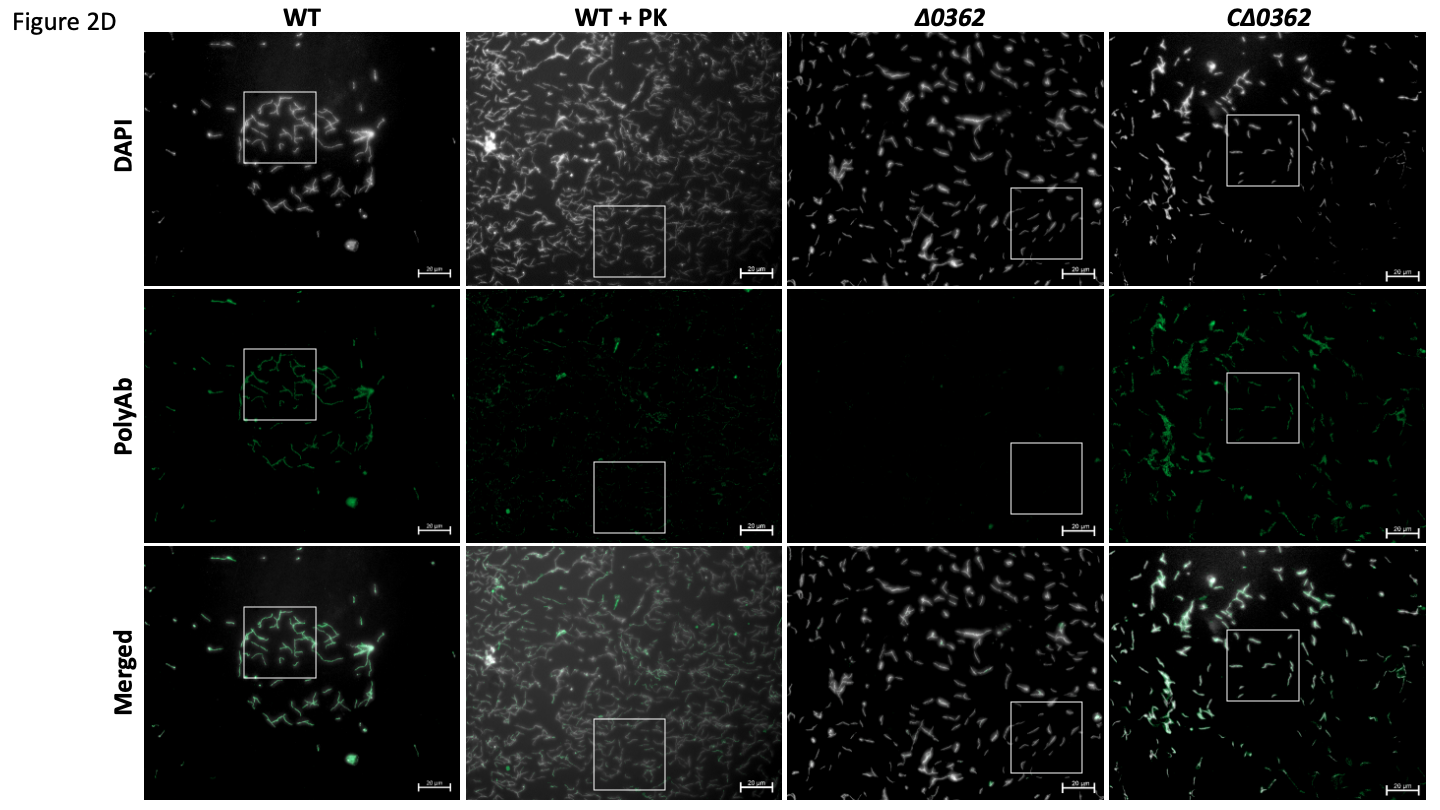

Supplement: Supplementary file 5 — Source data Fig. 2 [file 44318_2024_342_MOESM5_ESM.zip › Figure 2/Figure 2D.tiff]

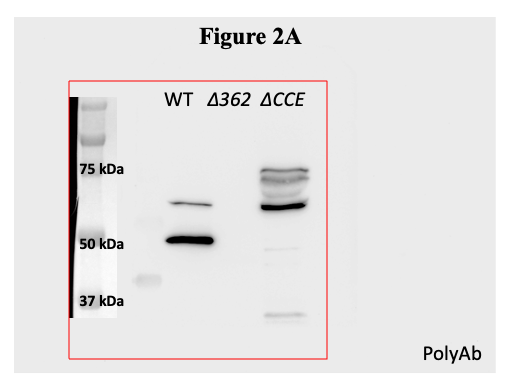

Supplement: Supplementary file 5 — Source data Fig. 2 [file 44318_2024_342_MOESM5_ESM.zip › Figure 2/Figure 2A.tiff]

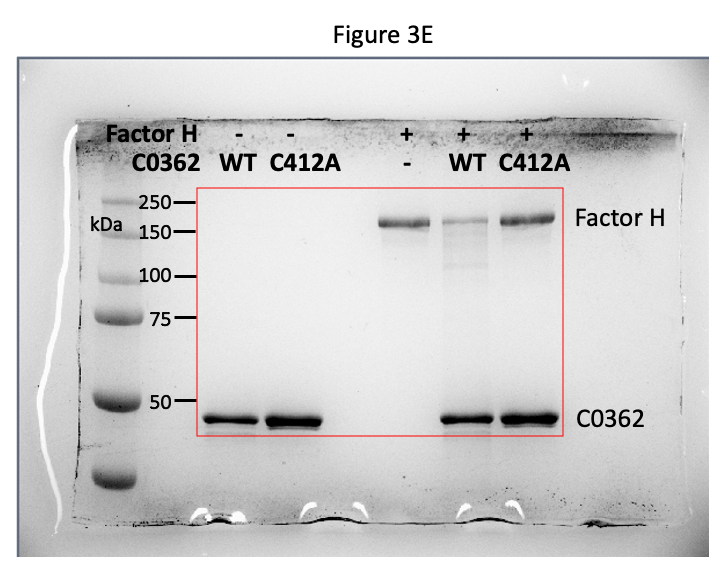

Supplement: Supplementary file 6 — Source data Fig. 3 [file 44318_2024_342_MOESM6_ESM.zip › Figure 3/Figure 3E.tiff]

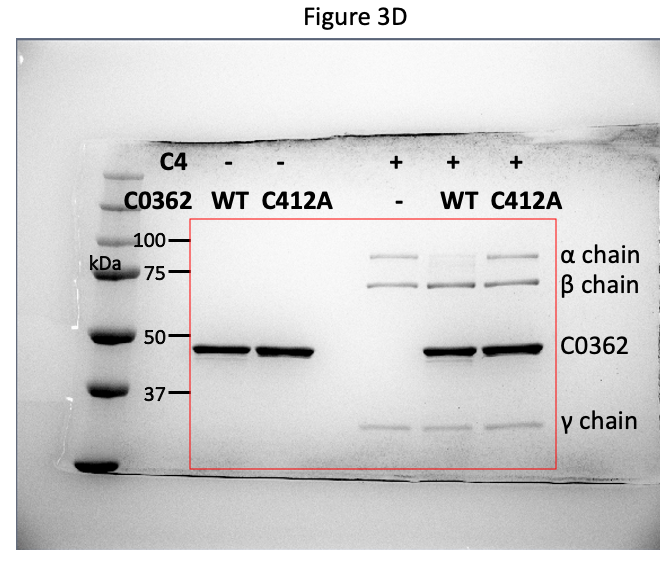

Supplement: Supplementary file 6 — Source data Fig. 3 [file 44318_2024_342_MOESM6_ESM.zip › Figure 3/Figure 3D.tiff]

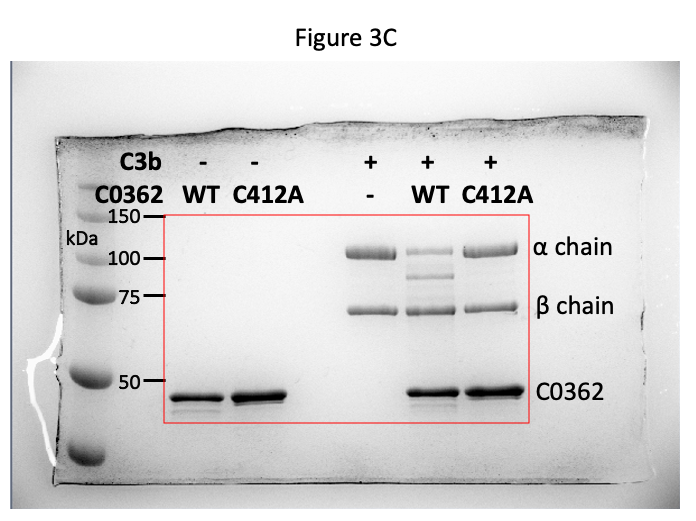

Supplement: Supplementary file 6 — Source data Fig. 3 [file 44318_2024_342_MOESM6_ESM.zip › Figure 3/Figure 3C.tiff]

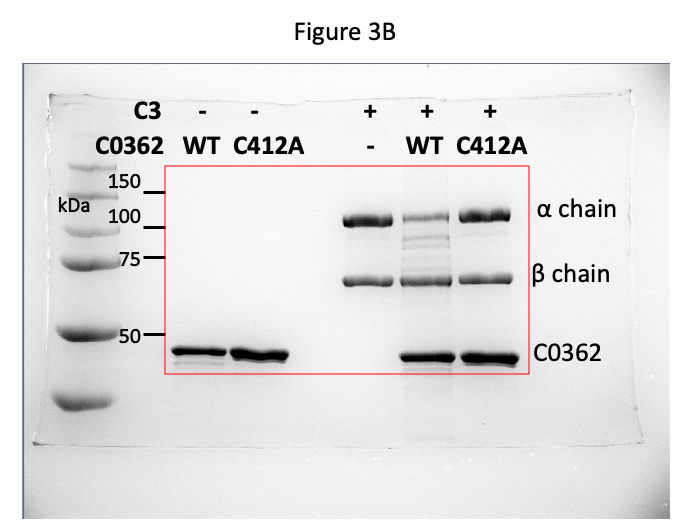

Supplement: Supplementary file 6 — Source data Fig. 3 [file 44318_2024_342_MOESM6_ESM.zip › Figure 3/Figure 3B.tiff]

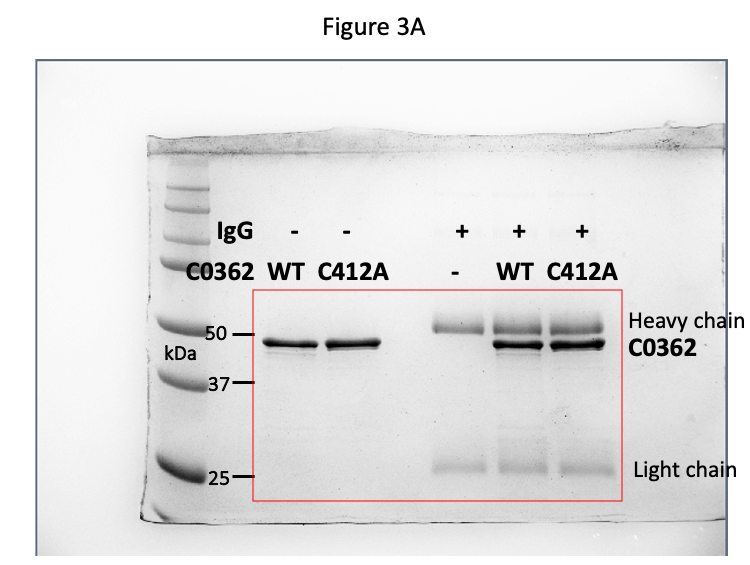

Supplement: Supplementary file 6 — Source data Fig. 3 [file 44318_2024_342_MOESM6_ESM.zip › Figure 3/Figure 3A.tiff]

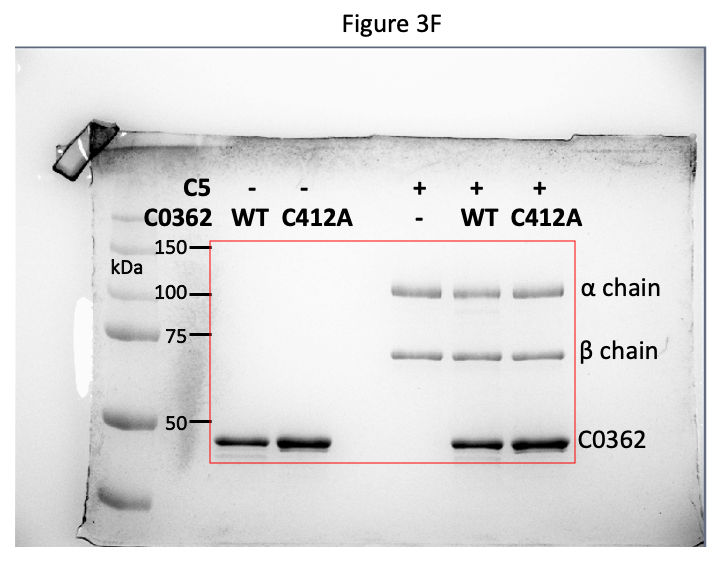

Supplement: Supplementary file 6 — Source data Fig. 3 [file 44318_2024_342_MOESM6_ESM.zip › Figure 3/Figure 3F.tiff]

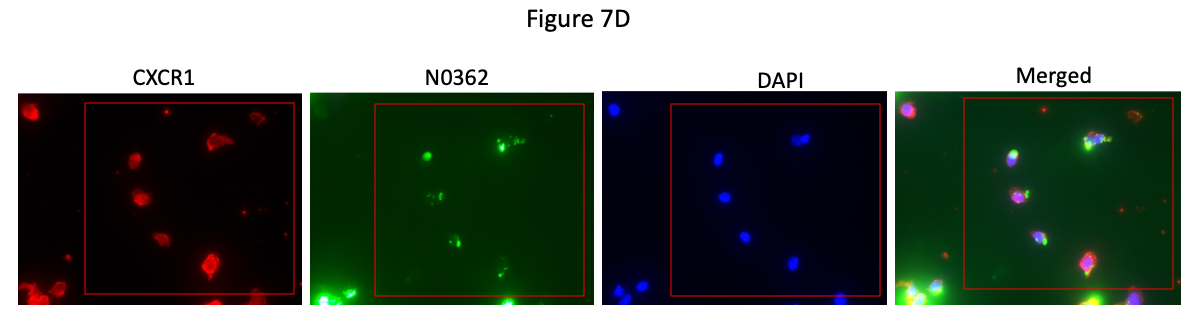

Supplement: Supplementary file 8 — Source data Fig. 7 [file 44318_2024_342_MOESM8_ESM.zip › Figure 7/Figure 7D.tiff]

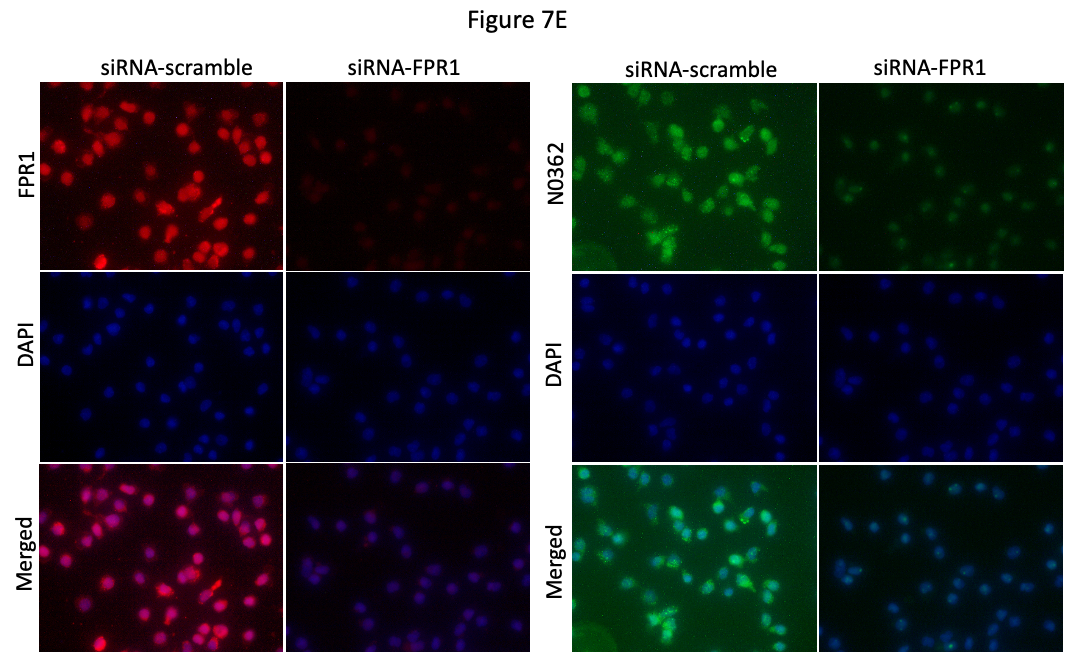

Supplement: Supplementary file 8 — Source data Fig. 7 [file 44318_2024_342_MOESM8_ESM.zip › Figure 7/Figure 7E.tiff]

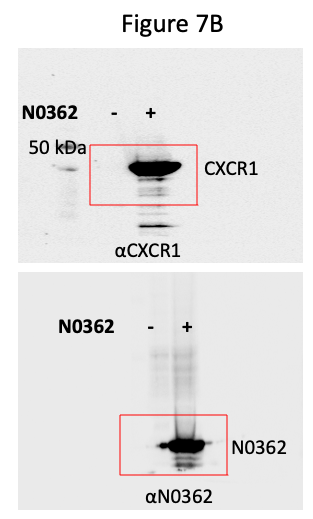

Supplement: Supplementary file 8 — Source data Fig. 7 [file 44318_2024_342_MOESM8_ESM.zip › Figure 7/Figure 7B.tiff]

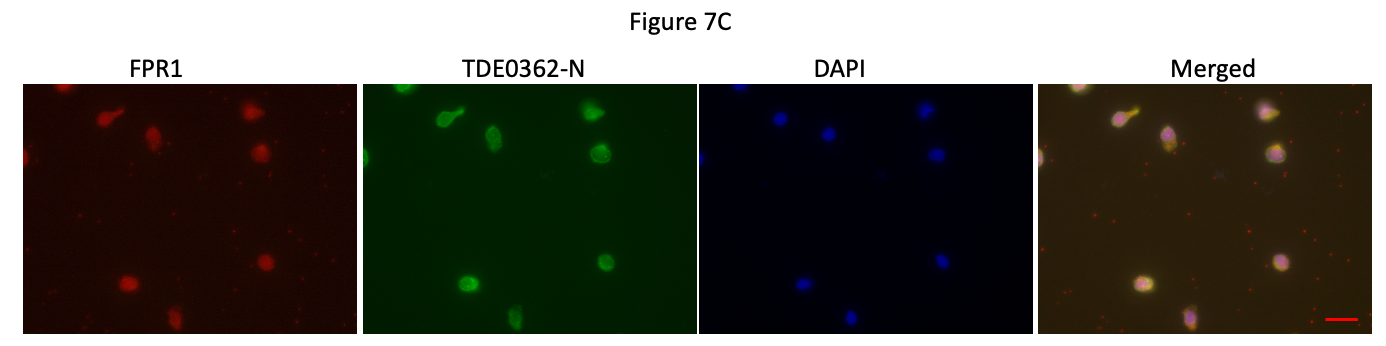

Supplement: Supplementary file 8 — Source data Fig. 7 [file 44318_2024_342_MOESM8_ESM.zip › Figure 7/Figure 7C.tiff]

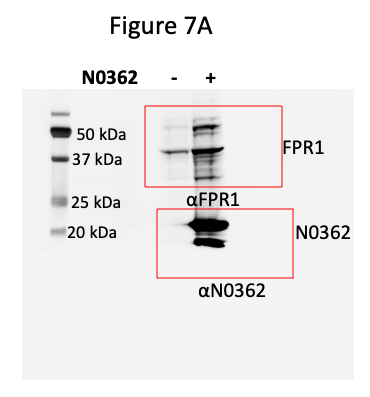

Supplement: Supplementary file 8 — Source data Fig. 7 [file 44318_2024_342_MOESM8_ESM.zip › Figure 7/Figure 7A.tiff]

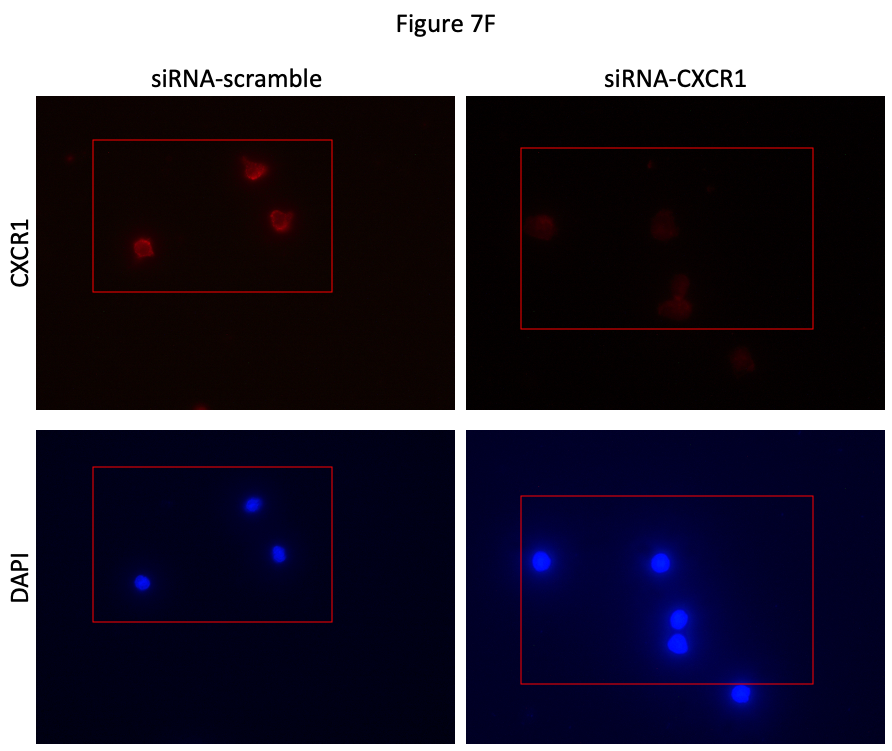

Supplement: Supplementary file 8 — Source data Fig. 7 [file 44318_2024_342_MOESM8_ESM.zip › Figure 7/Figure 7F.tiff]

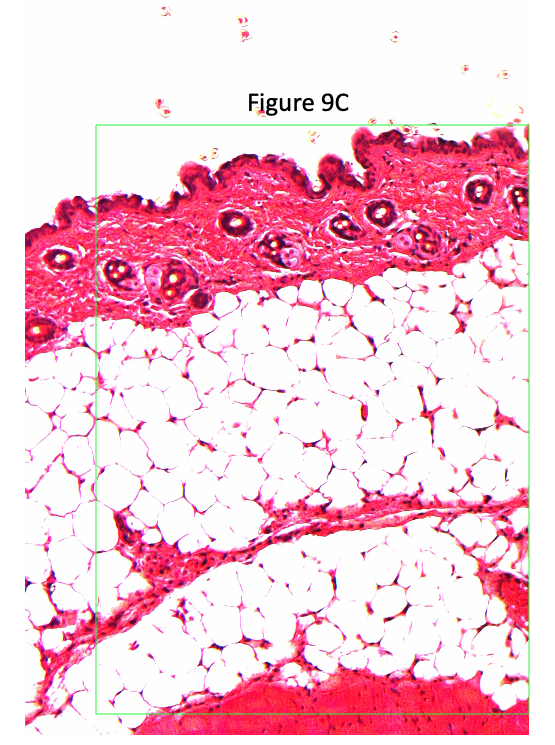

Supplement: Supplementary file 10 — Source data Fig. 9 [file 44318_2024_342_MOESM10_ESM.zip › Figure 9/Figure 9C.tiff]

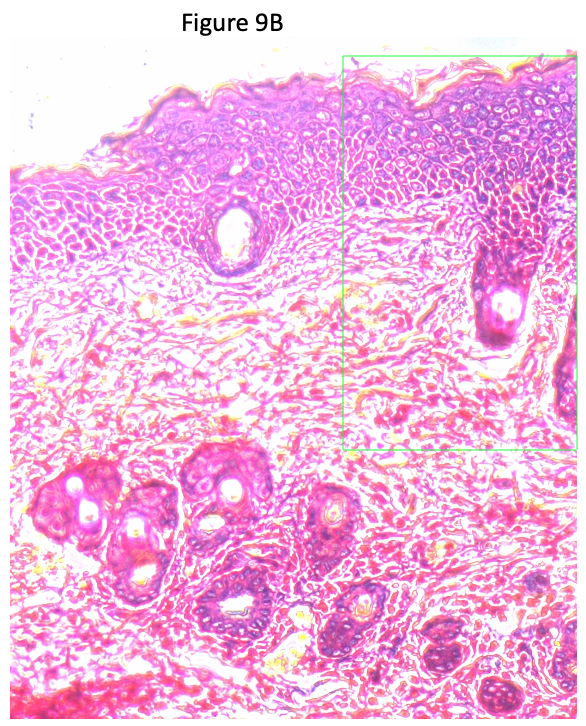

Supplement: Supplementary file 10 — Source data Fig. 9 [file 44318_2024_342_MOESM10_ESM.zip › Figure 9/Figure 9B.tiff]

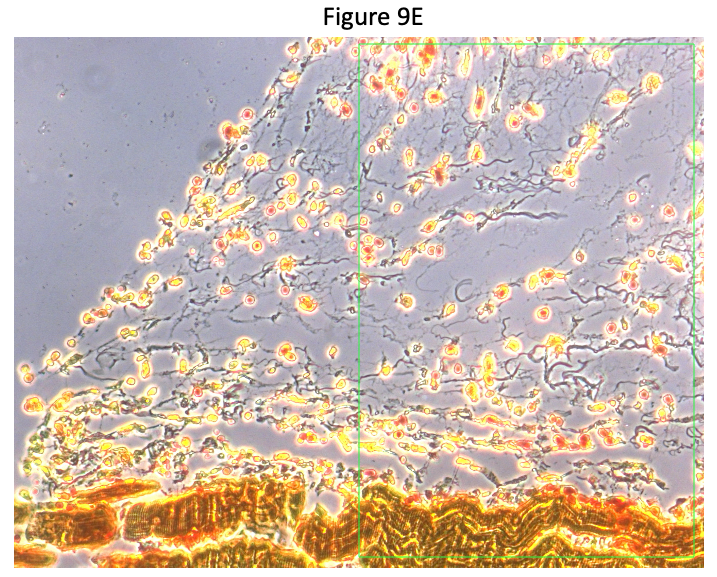

Supplement: Supplementary file 10 — Source data Fig. 9 [file 44318_2024_342_MOESM10_ESM.zip › Figure 9/Figure 9E.tiff]

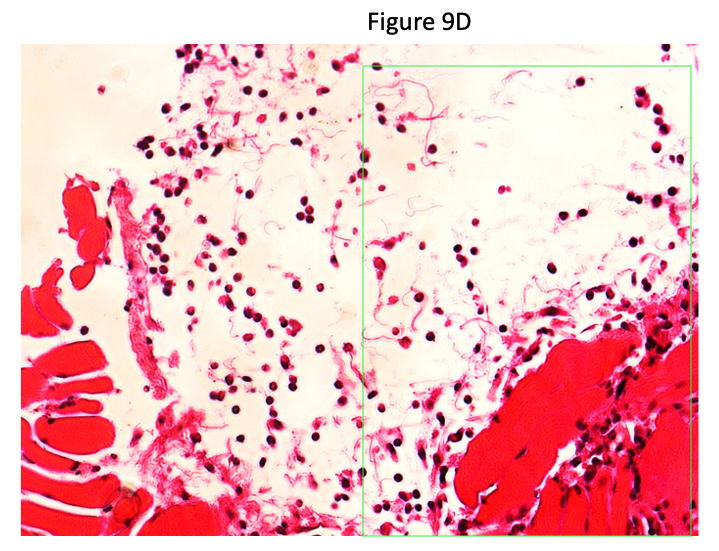

Supplement: Supplementary file 10 — Source data Fig. 9 [file 44318_2024_342_MOESM10_ESM.zip › Figure 9/Figure 9D.tiff]
