# Supplementary material for: A bipartite bacterial virulence factor targets the complement system and neutrophil activation
Source: EMBO J. 2025 Jan 3;44(4):1154–84. doi: 10.1038/s44318-024-00342-8 (PMC11833123; doi:10.1038/s44318-024-00342-8)
Supplement: Supplementary file 11 — Expanded View Figures [file 44318_2024_342_MOESM11_ESM.pdf]

Expanded View Figures

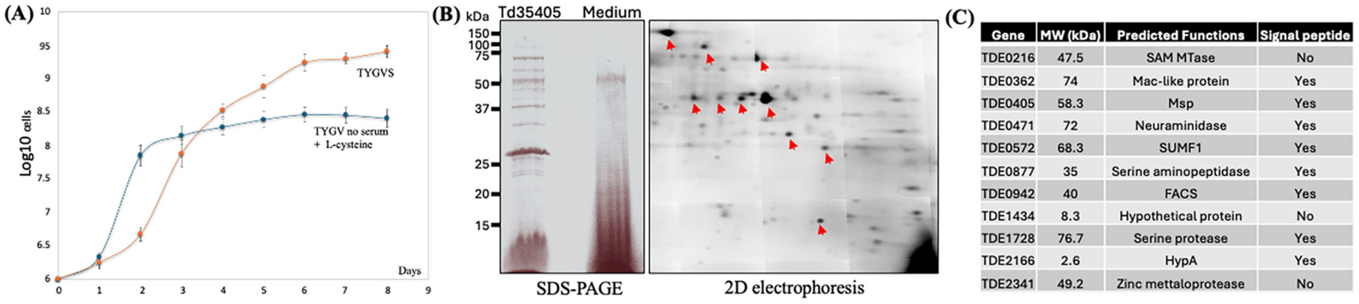

Gene	MW (kDa)	Predicted Functions	Signal peptide
TDE0216	47.5	SAM MTase	No
TDE0362	74	Mac-like protein	Yes
TDE0405	58.3	Msp	Yes
TDE0471	72	Neuraminidase	Yes
TDE0572	68.3	SUMF1	Yes
TDE0877	35	Serine aminopeptidase	Yes
TDE0942	40	FACS	Yes
TDE1434	8.3	Hypothetical protein	No
TDE1728	76.7	Serine protease	Yes
TDE2166	2.6	HypA	Yes
TDE2341	49.2	Zinc mettaloprotease	No

Figure EV1. Analysis of *T. denticola* ATCC35405 cell spent culture supernatants (SCSs).

(**A**) Growth curves of *T. denticola* ATCC 35405 in the TYGVS medium with or without rabbit serum supplemented with L-cysteine (1 g/liter). Cell counting was repeated in triplicate. (**B**) SDS-PAGE (left panel) and 2D-gel electrophoresis (right panel) of *T. denticola* SCSs prepared from the serum-free growth medium. Samples with the medium alone served as a negative loading control. The red arrows indicated 11 demarcated spots that were excised and subjected to matrix-assisted laser desorption/ionization-time of flight mass spectrometry (MALDI-TOF MS) analysis. (**C**) A list of 11 proteins were identified in *T. denticola* SCSs by MALDI-TOF MS with high confidence. Signal peptides (SPs) were predicted using Signal IP 5.0.

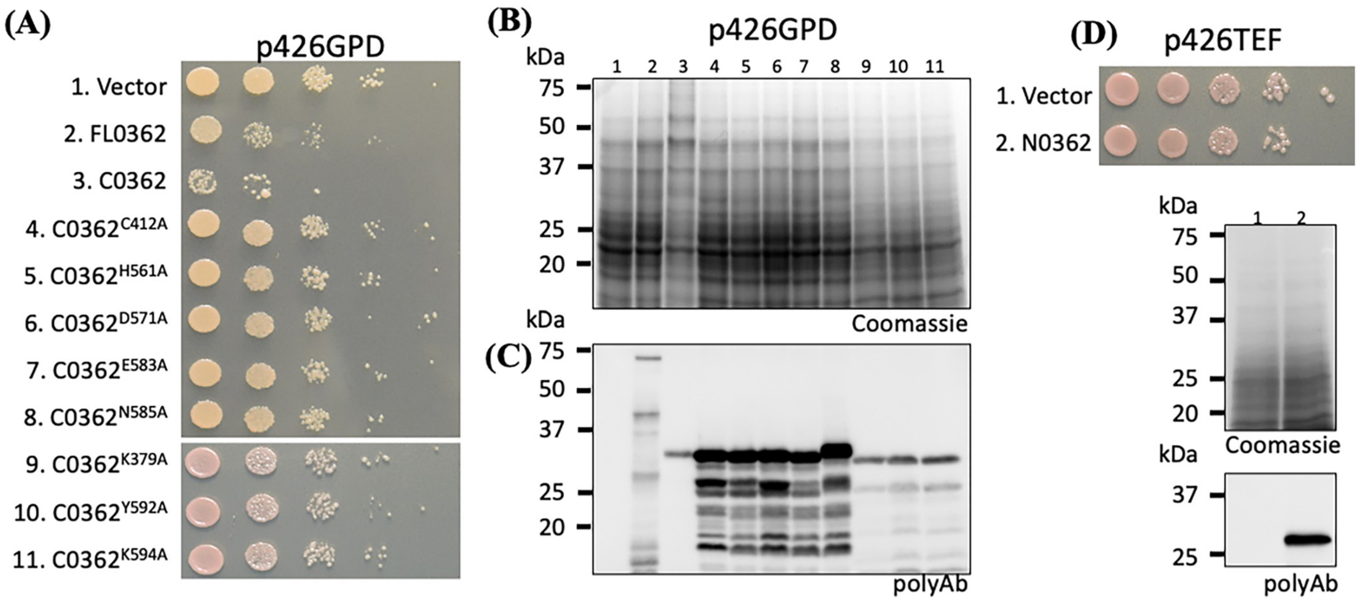

Figure EV2. Yeast cell toxicity assays.

(**A**) Yeast strains expressing the full-length TDE0362 (FL0362, 1–647 aa), C-terminal Mac-1 domain in TDE0362 (C0362, 331–647 aa), or eight point mutants as labeled, were expressed constitutively using GPD promoter (p426GPD). The strains were serial-diluted and spotted onto plates containing glucose. Plates were incubated at 30 °C for 48 h before image acquisition. (**B**, **C**) Monitoring the expression of TDE0362 proteins using SDS-PAGE followed by immunoblots using a specific polyclonal antibody against TDE0362 (polyAb). (**D**) N-terminal TDE0362 (N0362, 23–204 aa) was constitutively expressed in yeast cells using TEF promoter (p426TEF). The strains were serial-diluted and spotted onto plates containing glucose. Plates were incubated at 30 °C for 48 h before image acquisition. The expression of N0362 was monitored by immunoblots probed against polyAb.

 © The Author(s)

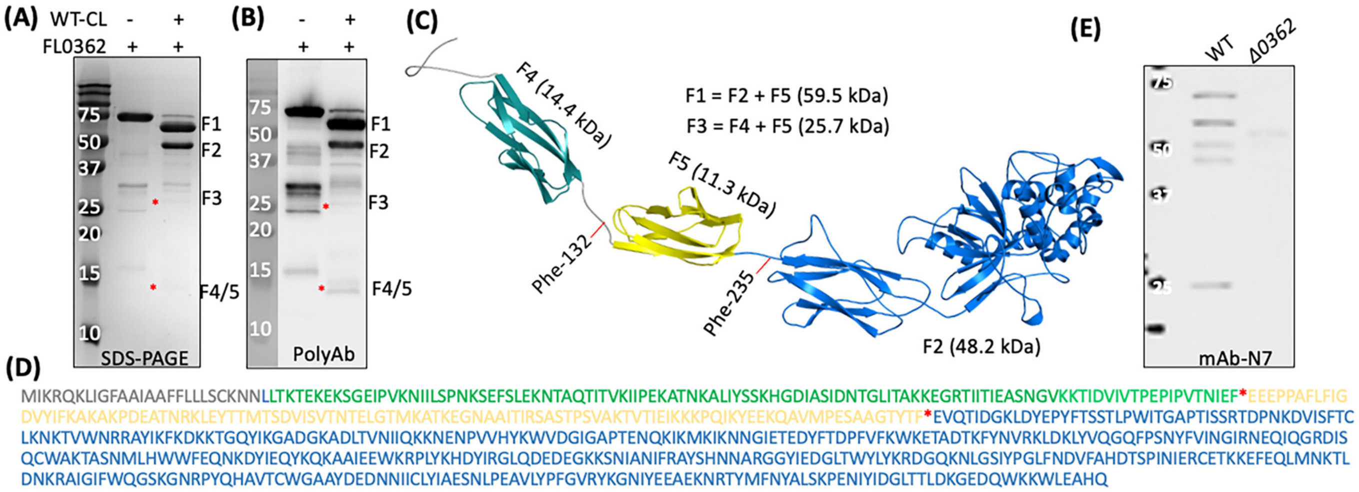

Figure EV3. Mapping TDE0362 cleavage sites.

For this study, the full-length TDE0362 (FL362) recombinant proteins were incubated with or without *T. denticola* WT cell lysate supernatants anaerobically for overnight; the resulted samples were subjected to SDS-PAGE (**A**) or immunoblotting probed against polyAb (**B**). The bands of F1 and F2 were excised and subjected to LC-MS/MS. Red asterisks are two minor cleaved bands. (**C**) A diagram illustrating the domain composition of TDE0362, cleavage sites, and the sizes of five cleaved products which were calculated based on their corresponding sequences. (**D**) The amino acid sequence of TDE0362. The detected cleavage products were labeled in different colors. * stands for the two Phe cleavage sites mapped. (**E**) Detection of TDE0362 in the SCSs of WT and *Δ0362* strains by immunoblots probed against mAb-N7, a monoclonal antibody against N0362. For this experiment, co-IP was first carried out to pull down TDE0362 cleaved products from the SCSs using polyAb and then probed against mAb-N7.

© The Author(s)

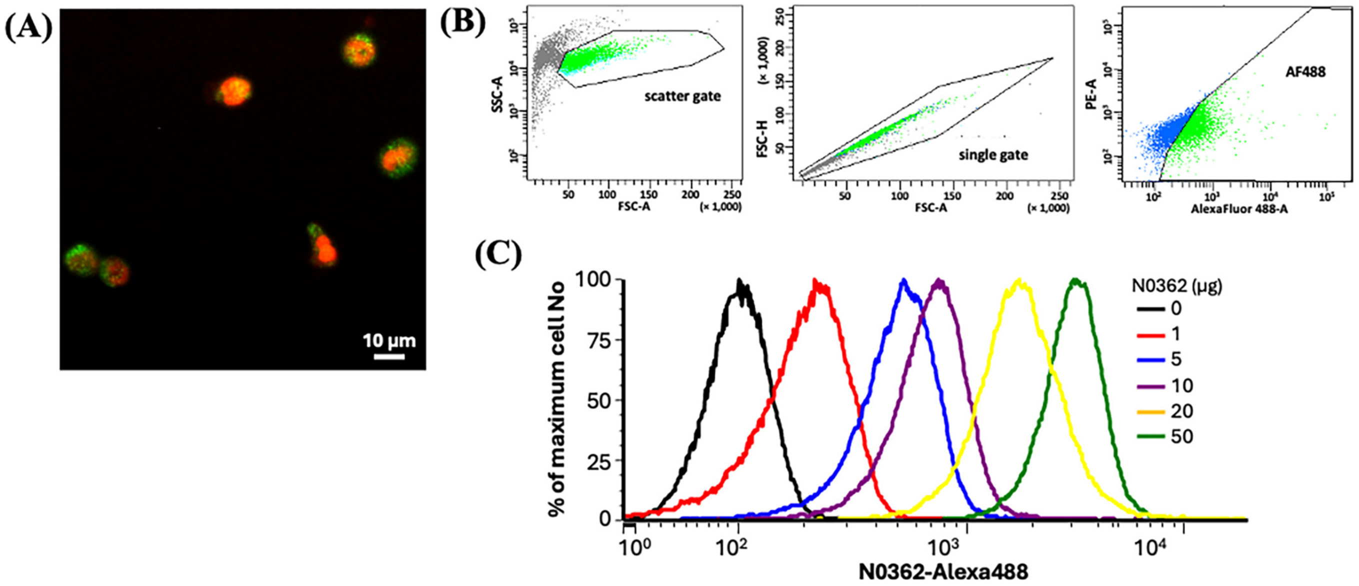

Figure EV4. N0362 binds to human neutrophils.

(**A**) Immunofluorescence staining using human neutrophil and Alexa Fluor 488 labelled N0362. The neutrophil was stained using propidium iodide. The scale bars represent 10 µm. (**B**, **C**) Flow cytometry was performed using human neutrophils and different concentrations of Alexa Fluor 488-labeled N0362, ranging from 0 to 50 µg of total proteins. (**B**) The gating cells strategy was based on FSC-A/SSC-A profile, followed by selecting single cells population (FSC-A/FCH-H), and subsequently detecting fluorescently labelled cells (Alexa Fluor 488-A/PE-A). (**C**) Histogram of flow cytometric analysis showed dose-dependent binding between neutrophil and N0362.

 © The Author(s)

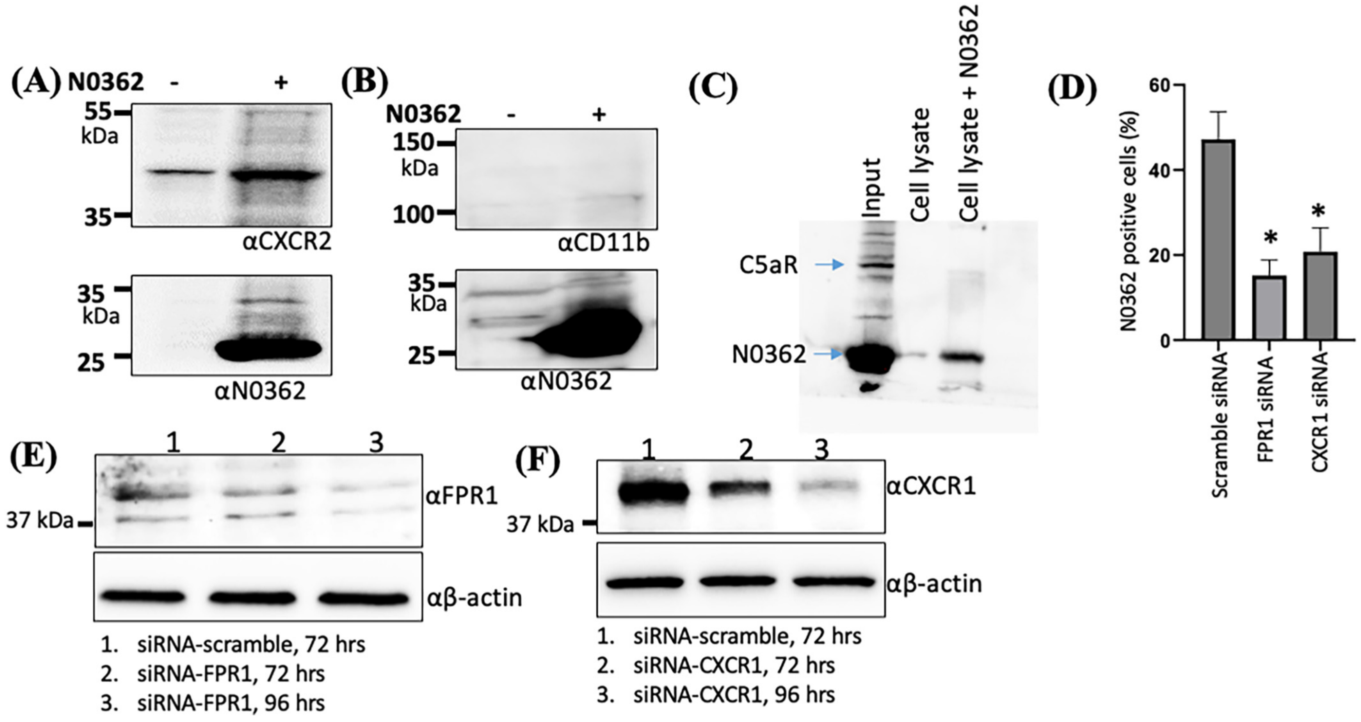

Figure EV5. Co-IP assays.

(**A–C**) For this experiment, DMSO-differentiated HL-60 cell (dHL-60) lysates were incubated with or without His-tagged N0362 protein (10 μg) for 3 h at 4 °C, followed by precipitation using Ni-NTA resin. The resulted co-IP samples were subjected to immunoblotting analysis probed against mAb-N7, a monoclonal antibody that recognizes N0362. (**A**) anti-CXCR2 (αCXCR2), (**B**) anti-CD11b (αCD11b), and (**C**) anti-C5aR (αC5aR). (**D**) siRNA knockdown of FPR1 or CXCR1 reduces the binding of N0362 to HL-60 cells. For this experiment, activated HL-60 cells were transfected with scramble siRNA (control), FPR1 siRNA or CXCR1 siRNA for 3 days and then the cells were co-incubated with His-tagged N0362 for 2 h. Cells were then fixed and stained anti-His antibody. Fluorescent cells were counted and recorded as percentage of positive cells in relative to total cells. **P* value < 0.05. (**E**, **F**) Detection of FPR1 and CXCR1 by immunoblotting analysis. Lane 1: scramble siRNA; Lane siRNA-FPR1 or CXCR1 72 h after the knockdown; and Lane 3: siRNA-FPR1 or CXCR1 96 h. β-actin was used as a loading control.

© The Author(s)
